# Epigenetic Feedback Regulation Accelerates Adaptation and Evolution

**DOI:** 10.1371/journal.pone.0061251

**Published:** 2013-05-08

**Authors:** Chikara Furusawa, Kunihiko Kaneko

**Affiliations:** 1 Quantitative Biology Center (QBiC), RIKEN, Suita, Osaka, Japan; 2 Research Center for Complex Systems Biology, University of Tokyo, Komaba, Meguro-ku, Tokyo, Japan; Instituto de Biología Molecular y Celular de Plantas, Spain

## Abstract

A simple cell model consisting of a gene regulatory network with epigenetic feedback regulation is studied to evaluate the effect of epigenetic dynamics on adaptation and evolution. We find that, the type of epigenetic dynamics considered enables a cell to adapt to unfamiliar environmental changes, for which no regulatory program has been prepared, through noise-driven selection of a cellular state with a high growth rate. Furthermore, we demonstrate that the inclusion of epigenetic regulation promotes evolutionary development of a regulatory network that can respond to environmental changes in a fast and precise manner. These results strongly suggest that epigenetic feedback regulation in gene expression dynamics provides a significant increase in fitness by engendering an increase in cellular plasticity during adaptation and evolution.

## Introduction

Cells adjust their internal states in order to adapt to environmental and genetic perturbations. Although such adaptive responses are generally described on the basis of specific if-then-like regulatory programs, for example, that provided by the lac operon [1], it is not always possible to account for the flexible adaptive dynamics observed in actual organisms. Indeed, it has been shown that microorganism strains with deletions of metabolic genes [2]–[4] or rewired regulatory networks [5], [6] can adapt to several environmental conditions, even though they have never experienced such perturbed conditions. Such adaptive dynamics displayed in response to unfamiliar environmental changes are difficult to account for regulatory programs fixed in advance. Hence, it would appear that there is some sort of generic, ubiquitous mechanism that enables cells to adapt to ever-changing environments [7]. Of course, cells can adapt quickly to environmental changes of a type that they have previously experienced frequently. It is necessary both to identify the mechanism that allows for adaptation to unfamiliar changes and to elucidate how this mechanism combines with the mechanism that allows for fast, precise adaptation to familiar changes.

In this study, we show that the combination of epigenetic regulation and gene regulatory dynamics enables cells to adapt to unfamiliar environmental changes and promotes the evolution of a fast and precise regulatory response. Recently, it has been demonstrated that epigenetic mechanisms based on several factors, including DNA methylation, histone modification, and their interplay with higher-order chromatin structure, play important roles in regulating and stabilizing the functional states of cells [8], [9]. These epigenetic regulation are suggested to form a positive feedback loop, which, together with gene regulation, may result in expression memory [10], [11]. Here, we introduce a simple model of such epigenetic feedback regulation(EFR) to investigate the effect of epigenetic dynamics on adaptation and evolution.

## Model

### 2.1 Expression Dynamics with Epigenetic Feedback

Let us consider a cell with a gene regulatory network consisting of 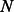 genes. The state of this cell can be represented by a vector of gene expression levels 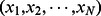, where 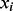 is the expression level of the 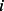-th gene. Gene expression refers to the synthesis of the corresponding protein, which may affect the expression of other genes, thus giving rise to a gene regulatory network. Noting that the expression of genes often exhibits on-off switching behavior, we adopt the following gene expression dynamics.
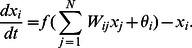
(1)


The first term on the right-hand side represents the synthesis of protein, while the second term corresponds to the degradation or dilution of protein. Here, 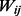 is the regulatory matrix. Its 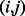-th element represents the regulation of the expression of the 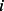-th gene exercised by the 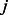-th gene. The elements of this matrix take the values 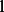, 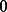, or 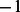, corresponding to activation, no regulatory interaction, and repression of the expression of the 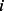-th gene by the 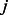-th gene, respectively. The synthesis of protein is represented by the sigmoidal regulation function 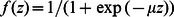, where 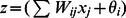 is the total regulatory input, and 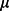 is the gain parameter of the sigmoidal function. If at some time the regulatory input for the 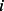-th gene is positive, then, under the dynamics described by Eq. (1), 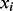 will rapidly increase toward 1 (the state of full expression), while if it is negative, it will rapidly decrease toward 0 (the state of non-expression). The regulatory interactions 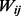 are chosen randomly from a distribution such that the probabilities that the 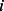-th gene activates or represses the expression of the 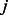-th gene are given by some value 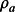 and 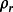, respectively.

The variable 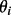 represents the epigenetic control of the expression of the 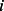-th gene. A positive value of 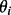 promotes the expression of the i-th gene, while a negative value represses it. In this study, we assume that the change in the epigenetic control factor 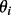 is determined by the expression level of the corresponding gene: The tendency for 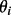 to increase (decrease) becomes stronger as 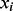 increases (decreases), i.e., positive feedback between the expression levels and the epigenetic control arises. We also assume that this EFR depends on the cellular activity, such as metabolic activity and protein synthesis activity, or individual cellular growth rate. The variable 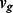 represents such growth activity. Furthermore, we assume that the dynamics of the epigenetic factors are noisy, due to their dependence on chemical reactions with a small number of molecules. Based on these assumptions, the time evolution of the 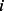-th epigenetic factor is modeled as follows:
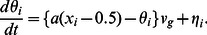
(2)


The parameter 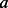 is a positive constant representing the strength of the epigenetic feedback control. The quantity 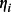 represents the stochastic part of the epigenetic dynamics and is assumed to be Gaussian white noise of amplitude 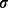. The cellular growth rate 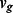 is assumed to be determined by the distance of the expression levels of 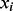 of a subset of 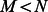 genes from a given target expression pattern 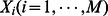:
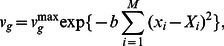
(3)where 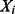 is the target expression level of the 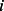-th gene, 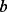 is a constant parameter, and 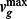 represents the maximal growth rate. The idea is that these 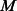 genes are directly involved in cellular activity, whereas the remaining 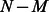 genes have only regulatory function.

### 2.2 Evolutionary Simulation of Regulatory Networks

We carried out evolution simulation of regulatory networks in which those exhibiting higher growth rates under 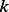 environments were selected. The target expression pattern of the 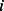-th gene in the 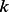-th environment, 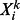, determined the growth rate. This target pattern was chosen randomly in the simulation. First, we prepared 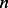 randomly generated regulatory networks as the parent networks. From each parent network, 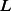 mutant networks were generated by randomly replacing a single reaction path to the parent network. Then, by using these mutant networks, the growth rates under 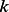 environmental conditions were calculated. Among such networks the top 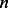 networks with regard to the geometric average of growth rate 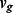 were selected as the parent networks for the next generation. By iterating this process, networks with high fitness values were obtained, i.e., networks that exhibited with higher growth rates under multiple environments. In this simulation, a common expression pattern determined randomly is used as the initial expression pattern in each environment and each generation.

## Results

### 3.1 Noise-driven Adaptation with EFR

**Figure 1:**
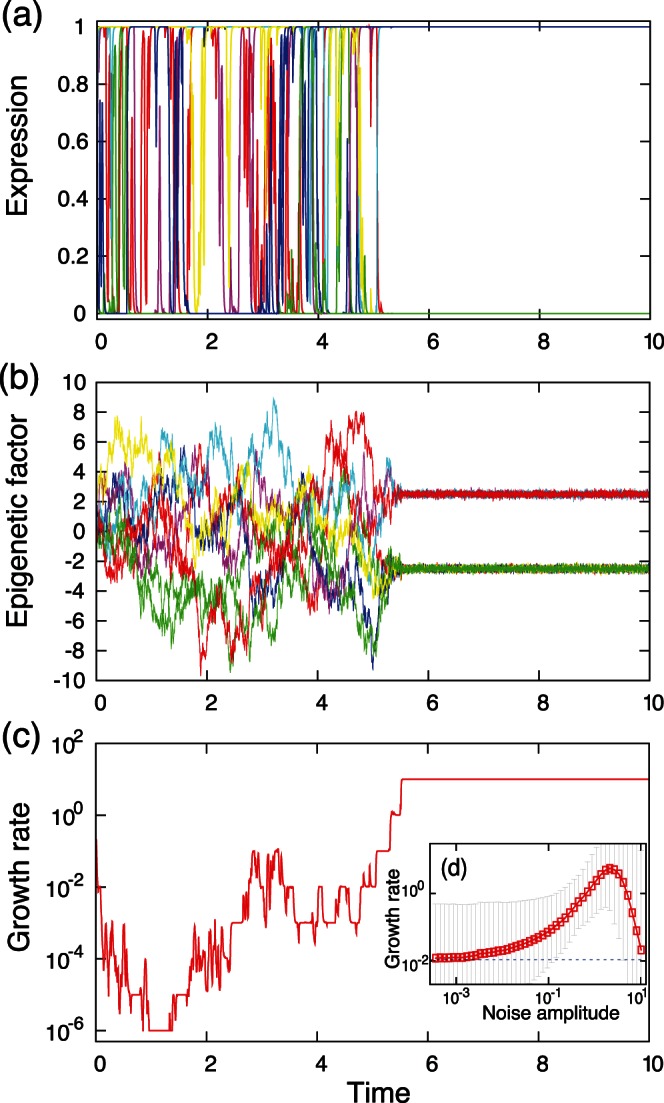
An example of adaptation process with EFR. (a) Time series of expression levels 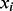. Eight of the 40 gene expression levels are displayed.(b) Time series of the epigenetic factors 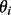 that correspond to the expression levels displayed in (a). (c) Change in the growth rate 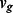. (d) (inset of (c))The relationship between the noise amplitude 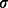 and the growth rate 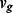. The geometric mean of the growth rates attained by randomly generated regulatory networks and initial conditions is plotted as a function of the noise amplitude 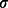. The error bars represent the geometric standard deviation. The blue dotted line indicates the growth rate in the case of a random selection of the state, where the expression level 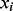 takes the values 0 and 1 with equal probability. The growth rate was significantly higher than the random expression pattern, for example 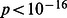 when 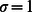 (the number of data was 10000; determined by U-test). The parameter values used here are 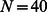, 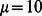, 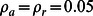, 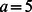, 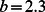, 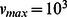, 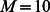, and 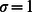. The target expression patterns 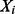 used in the growth rate calculation were determined randomly. Unless otherwise mentioned, these values were used throughout all the figures. The presented results were independent of specific model parameters, and were observed in a wide range of parameter values. We selected above parameter values to present general features of this model.

**Figure 2:**
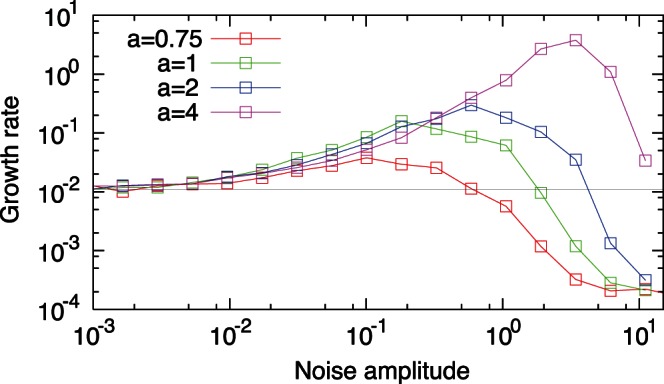
The relationship between the noise amplitude 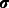 and the growth rate 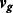 with various feedback strengths. The geometric mean of the growth rates attained by randomly generated regulatory networks is plotted as a function of the noise amplitude 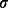. The growth rates for different values of 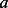 are superimposed by using different colors. The black line indicates the growth rate in the case of a random selection of the state, where the expression level 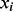 takes randomly the values 0 and 1 with equal probability.

We carried out numerical simulations with the model described above using several sets of parameter values and thousands of randomly generated regulatory networks, and found that adaptation dynamics that result in an actively growing state generally emerge with the aid of EFR. Fig. 1 depicts a typical example of such an adaptation process with the noise amplitude 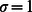. Time series of the expression levels of arbitrarily chosen genes, their epigenetic control factors, 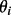, and the growth rate, 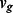, are plotted in Figs.1(a)–(c), respectively. In this example, the cell is initially placed in a state with a low growth rate, where the time evolution of the 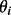 is characterized by large-amplitude fluctuations, due to the noise term in Eq. (2), and with this fluctuation of 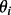, the expression levels exhibit switching dynamics between 0 and 1 (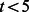 in Fig. 1). However, after itinerating among various expression patterns, the cell eventually settles into a state with a high growth rate, which is maintained over time (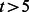 in Fig. 1). When 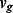 is small, the deterministic part in the dynamics of 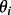 is small, and thus the stochastic part in Eq. (2) dominates the epigenetic dynamics, resulting fluctuating dynamics of both gene expression and epigenetic factors. By contrast, when the expression pattern approaches the target pattern through such fluctuations, and thus 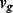 becomes large, the epigenetic feedback mechanism begins to regulate the expression dynamics. Then, if 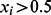, 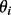 will converge to some positive value, and 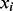 converges to 1, while if 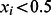, 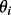 will converge to some negative value, and 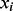 converges to 0. In either case, the resulting states of 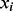 and 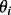 are mutually stabilizing. With this growth-dependent feedback control of expression levels through the dynamics of 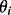, the expression pattern is ''memorized'' by the values of the epigenetic factors, but only when 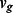 is large. Consequently, the network spontaneously converges to an expression pattern characterized by a high growth rate. Fig. 1(d) shows how the noise amplitude determines the average growth rate. There, we plotted the geometric mean of the asymptotically realized growth rates as functions of the noise amplitude, obtained from 10,000 simulations employing randomly generated regulatory networks. It is seen that for a small noise amplitude, the growth rate remains at the value realized through random selection of the cellular state, because a cell cannot escape from the attracting state that it first reached. Contrastingly, when the noise amplitude is very large (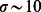), the growth rate is small, because the cellular state continues to itinerate due to the influence of the noise, settling into no attracting state. In the intermediate range, cells realize relatively high growth rates, through EFR and noise. We confirmed that the results are independent of the choice of the initial condition (i.e., gene expression levels and epigenetic factors). Fig. 2 shows how the growth rate depends on the strength of epigenetic feedback control 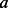. We confirmed that the noise-driven adaptation is observed when 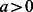.

### 3.2 An Alternative Model: EFR without Growth Dependency

**Figure 3:**
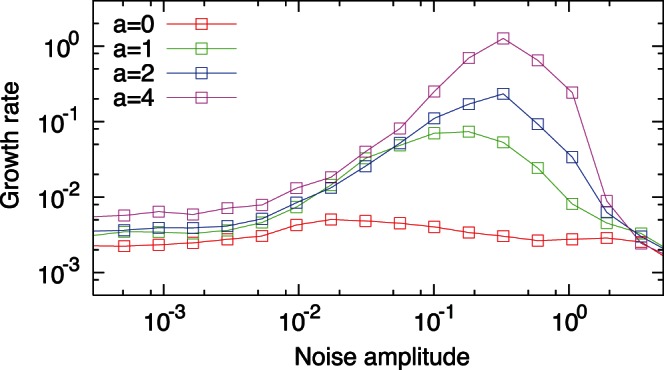
The relationship between the noise amplitude 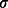 and the growth rate 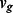 obtained by the alternative model without the growth-dependence on EFR. The geometric mean of the growth rates attained by randomly generated regulatory networks is plotted as a function of the noise amplitude 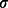. The growth rates for different values of 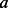 are superimposed by using different colors. In the intermediate range of the noise amplitude 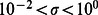, the average growth rates obtained in the cases with 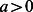 are significantly higher than the case without EFR (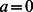), due to the selection of actively growing state by noise.

In the model presented above, we assumed that the EFR depends on the growth rate (activity) 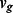. However, this assumption is not necessary for the adaptive dynamics with the aid of EFR. To demonstrate it, here we have constructed an alternative model with EFR in which only the gene expression dynamics depends on growth activity, 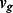, and expression noise, while the epigenetic factor 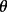 depends only on the levels of itself and corresponding gene expression. As a specific example, we studied the following model:
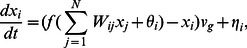
(4)

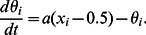
(5)where 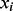 is the expression level of the 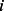-th gene, 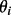 represents the epigenetic control for it, 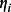 is Gaussian white noise, and 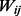 is regulation matrix, as in Eq. (1). Eq.(4) with fixed 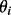 is almost identical to our previous model [7]. Here, when the growth rate is small, the stochastic part in Eq.(4) is dominant in the expression dynamics to enable a cell to search a variety of expression states. Then, when the expression dynamics falls into an attractor with active growth, the deterministic part (the first term of the left-hand side in Eq. (4)) starts to be dominant in the expression dynamics by which the actively growing state is maintained. In this mechanism, when there is no epigenetic feedback (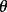 is fixed to zero), the expression dynamics itself need to prepare a large number of attractors to maintain the adaptive dynamics, as discussed in our previous study. In contrast, by introducing epigenetic feedback (Eq.(5)), the selection of an actively growing state is possible even when the expression dynamics themselves possess a small number of attractors. In Fig. 3, we present how the noise amplitude determines the average growth rate in this model. Here, EFR supports to stabilize the expression states with a high growth rate 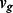. Here, EFR stabilizes expression states of a high growth rate, 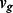. It should also be stressed that this result demonstrates the generality and robustness of adaptive dynamics with our proposed EFR. In fact, we tested several models with EFR, and found that the selection of an actively growing state is possible in all.

### 3.3 Acceleration of Network Evolution by EFR

**Figure 4:**
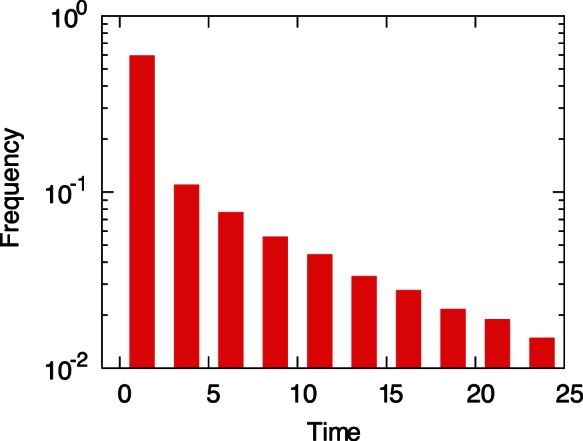
The distribution of search time. Starting from random initial condition, the time to reach a steady expression pattern was calculated. For 4.1% of trials, the search time was larger than 25 unit time. The model and parameter values were identical to those in Fig. 1.

The effectiveness of the mechanism of adaptation discussed above requires no fine-tuning of the parameters and the regulatory networks, and allows the cell to adapt to environmental changes that it has not experienced in the course of evolution. Of course, there are demerits to this mechanism, too. Most significantly, it is inefficient; that is, the time required for adaptation, in comparison with the unit time in Eq.(1), is relatively long. Fig. 4 shows the distribution of search time needed for the expression dynamics to reach a steady state by starting from random initial condition, where the median of search time is at around 3.9 unit time. This indicated that, before settling into a stable expression pattern with a high growth rate, the cell generally need to search more than one hundred on-off expression patterns. This inefficiency, however, is due to the randomly generated nature of the regulatory networks. The regulatory networks of actual organisms have been organized through evolution. These networks allow for quick, precise responses to environmental changes of types already experienced.

**Figure 5:**
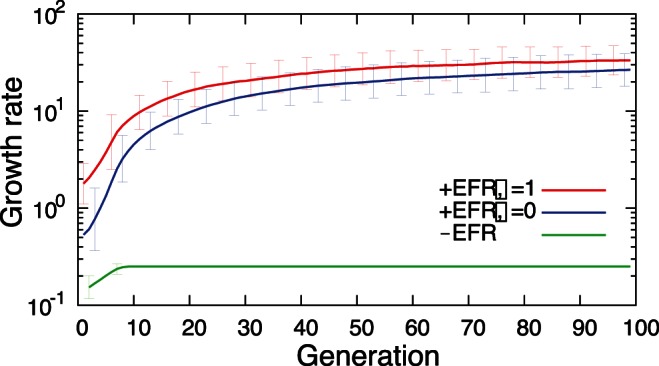
Evolution of the highest fitness as a function of the generation. 100 independent evolutionary simulations were performed, and the average of the highest fitness value in each generation is plotted along with the standard deviation. The fitness of a regulatory network is defined as the geometric average of the growth rate, 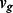, in 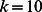 different environments. For the cases with EFR, the noise amplitude, 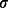, was set to 0 and 1, respectively. For each generation, the number of parent networks 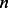 was set to 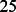, while for each parent network 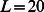 mutant networks were generated by randomly replacing a regulatory path. The total number of regulatory paths was fixed to 400. In the cases without EFR, the epigenetic factor 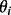 was fixed to 0.

Hence, we study the evolutionary dynamics of regulatory networks in order to investigate how this evolution is affected by the presence of EFR, by using the model described by Eq.(1) and (2). For this purpose, we define the fitness of a cell to be the geometric mean of the cell's growth rates in multiple environments realized after a relatively short period (one unit time in Fig. 1). Fig. 5 shows the increase in the highest fitness (i.e., average growth rate under multiple environments) among the mutant networks averaged over 100 independent evolutionary simulations with and without EFR (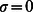 and 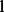). In this simulation, there is no environmental signal except for the change of the target expression patterns. Therefore, the cells without EFR cannot respond to the environmental changes and the fitness level is kept relatively low. In contrast, with EFR, the fitness levels increase with the evolution of the regulatory networks, regardless of the addition of noise. In such cases, the cell falls into an actively growing state under a given environmental condition without searching many on-off expression patterns as in Fig. 1(a), through the positive feedback of the epigenetic factors and the growth activity. Since EFR allows the cell to sense the environmental conditions, the evolutionary process then accelerates environmental adaptation through growth regulation, even without the aid of noise or environmental signals. It should be noted that, when environment-specific input signals are included appropriately, the fitness level increases even without EFR, but not so much unless the signal is very strong. The results indicate that, with the aid of EFR, the regulatory networks after evolution with a moderate noise level (e.g., 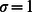) possess two advantageous properties, i.e., fast adaptation to known environments to which they are tuned, and noise-based adaptation to unknown environments.

**Figure 6:**
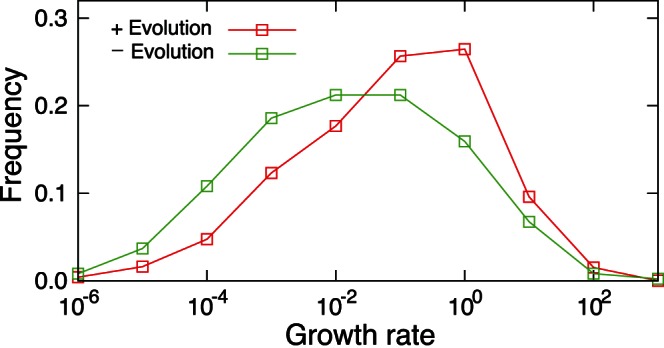
The distributions of growth rates in a novel environment for evolved networks and random networks. 100 evolved networks obtained after 100 generations with EFR (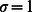; see Fig. 2) and 100 random networks were used for the calculations. For each network, the growth rates were calculated using 50 randomly chosen target expression patterns that were not used for the evolutionary simulations. For all simulations in this figure, 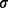 is set to 1 and EFR is incorporated in the expression dynamics. The difference in the growth rates was statistically significant (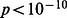; determined by U-test).

It is worth noting that the evolved networks obtained under specific environmental conditions exhibit higher growth rates in unfamiliar environments than the random network, as shown in Fig. 6. This is presumably due to the increase of sensitivity to perturbations, such as slight changes in regulatory inputs and expression noise, in the expression dynamics of evolved networks. To evaluate the sensitivity to perturbations in the expression dynamics, we calculated the Euclidean distance between two expression profiles 
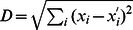
, where 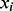 and 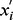 represent expression levels at steady states obtained by adding constant perturbations to the total regulatory input 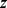, i.e., with addition of Gaussian noise with amplitude 0.001. The average distances over 100 evolved and random networks are 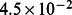 for the evolved networks and 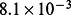 for random networks, respectively, suggesting a higher sensitivity to perturbations in the evolved networks than the random networks. This higher sensitivity maintained in the evolved networks can accelerate the noise-driven search for a higher-growth state. Also, we confirmed that the average time required to reach a steady state in unfamiliar environments is significantly shorter in the evolved networks than the random networks. This is another reason why the evolved networks achieve higher fitness in unfamiliar environments.

## Discussions

In this study, we have proposed a model describing the dynamics of gene regulatory networks possessing EFR. We have shown that the existence of EFR enables cells to adapt to unfamiliar environmental changes through a stochastic selection of expression patterns that support a high growth rate. Under the presence of EFR, evolution to achieve quick and precise adaptation to multiple environments is realized without environment-specific signals. We emphasize that the results presented herein are valid over a broad class of models, as long as there exist the following three characteristics. First, gene expression levels and corresponding epigenetic regulations form positive feedback loops. That is, when the expression level of a gene becomes high, the epigenetic feedback regulation is activated which further activates the expression, and vice versa. Second, the activity of EFR (and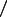) or gene expression dynamics somehow depends on the cellular activity as the growth rate. In the model represented by Eqs.(1) and (2), this growth dependency is included by assuming the change of the epigenetic factor 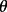 over time is proportional to the growth rate 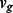. Alternatively, the feedback from the growth rate to gene expression dynamics allow for the present adaptation. In the model represented by Eqs. (4) and (5), the feedback is included by assuming that the protein synthesis and degradation rate are proportional to the growth rate. Finally, the dynamics of EFR (and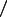) or gene expression dynamics are noisy, due to their dependence on chemical reactions with a small number of molecules. The noise can be introduced to the dynamics of epigenetic factor as in Eq.(2), or to the gene expression dynamics as in Eq.(4), or both. We have tested several models with the above characteristics, and confirmed that the noise-driven adaptation by EFR is generally observed. For example, we have confirmed that the noise-based adaptation we proposed also emerges when the cellular growth has a more complex dependency on the expression profile as adopted in [7], instead of the simple additive fitness function in Eq. (3). Furthermore, we have confirmed that the model with a Hill function form of gene regulation also exhibit the noise-driven adaptation. Indeed, these results suggested the generality of the adaptive dynamics with the aid of EFR. We also studied the relevance of the noise-driven adaptation with EFR under fluctuating environments. Numerically, the change of environmental conditions was introduced as the change of the target expression levels in Eq.(3) per some generations. When the time scale of environmental switches was longer than time required for adaptation, the cells maintained high growth rates if there existed appropriate level of the EFR (e.g. 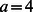 and 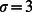 in Eqs. (1)(2)). The noise-driven adaptation works under continuously fluctuating environments.

Now we discuss molecular basis of EFR we proposed here. First, there are several studies that suggest the existence of feedback regulation in epigenetic dynamics. For example in bacterial cells, it is suggested that methylation of genomic DNA acts as an epigenetic mechanism with positive feedback regulation, which enables inheritance of DNA methylation patterns [12]. DNA supercoiling in bacterial genome is also known as an epigenetic factor which control the gene expression profile with positive feedback regulation. It is suggested that, the binding of RNA polymerase to genomic DNA promote the formation of negative supercoiling, while the formation of supercoiling accelerates the RNA polymerase binding [13]. Here, a positive feedback regulation of the transcription and formation of supercoiling arises, which results highly transcribed regions in the genome mainly corresponding to rRNA synthesis. It is natural to expect that such positive feedback regulation in DNA structure and transcription can maintain heritable epigenetic state. For examples of epigenetic feedbacks in eukaryote, a polycomb-based epigenetic switch in *arabidopsis* analyzed experimentally by [14] agreed with the theoretical model for positive feedback regulation presented by [10], [11]. Indeed, there are a number of studies addressing ''epigenetic memory" including one that shows gene expression is memorized by epigenetic mechanisms such as DNA and histone modifications [9], [15], [16]. To generate and maintain such memory, a positive feedback between gene expression and epigenetic mechanisms are expected to exist. Also, it may be that epigenetic feedback can be accelerated by an increase in the growth rate, because the numbers of RNA polymerase and other proteins involved in the epigenetic dynamics per cell are positively correlated with the growth rate [17]. In fact, in *E.coli* cells, the formation of highly transcribed regions by the positive feedback via DNA supercoiling is positively correlated with the cellular growth rate [13]. Furthermore, epigenetic dynamics contain noise, because gene expressions and other chemical reactions are inherently stochastic [18]–[21]. In fact, there are experiments supporting the notion that switches between epigenetic states are stochastic [22], . Along with the results presented here, these previous reports lead us to hypothesize that the epigenetic feedback mechanism underlies the flexible and robust dynamics of adaptation and evolution exhibited by living organisms.
